# Investigating the internal structure of multiple mini interviews—A perspective from Pakistan

**DOI:** 10.1371/journal.pone.0301365

**Published:** 2024-04-11

**Authors:** Rukhsana Ayub, Naveed Yousuf, Nadia Shabnam, Muhammad Azeem Ashraf, Azam S. Afzal, Ayesha Rauf, Danish Hassan Khan, Faiza Kiran

**Affiliations:** 1 Department of Health Professions Education, National University of Medical Science, Rawalpindi, Pakistan; 2 Department for Educational Development, The Aga Khan University, Karachi, Pakistan; 3 Research Institute of Education Science, Hunan University, Changsha, China; 4 Department of Community Health Sciences & Department for Educational Development, The Aga Khan University, Karachi, Pakistan; 5 Clinical Project Manager, Tiger Med Consulting Pakistan Ltd, Punjab, Pakistan; University of Valencia: Universitat de Valencia, SPAIN

## Abstract

**Background:**

Healthcare professionals require many personal attributes in addition to cognitive abilities and psychomotor skills for competent practice. Multiple Mini- Interviews are being employed globally to assess personality attributes of candidates for selection in health professions education at all level of entry; these attributes are namely, communication skills, critical thinking, honesty, responsibility, health advocacy, empathy and sanctity of life. Considering the high stakes involved for students, faculty, institutions and the society, rigorous quality assurance mechanisms similar to those used for student assessment must be employed for student selection, throughout the continuum of medical education. It is a difficult undertaking as these psychological constructs are difficult to define and measure. Though considered to yield reliable and valid scores, studies providing multiple evidences of internal structure especially dimensionality of Multiple Mini-Interviews are sparse giving rise to questions if they are measuring a single or multiple constructs and even if they are measuring what they are purported to be measuring.

**Objective:**

The main objective is to provide statistical support of the multi-dimensional nature of our Multiple Mini Interviews, hypothesized a*-priori*, through CFA. Another objective is to provide multiple evidences for the internal structure. Our study highlights the link between content and internal structure evidences of the constructs, thus establishing that our Multiple Mini Interviews measure what they were intended to measure.

**Method:**

After securing permission from the Institutional review board, an a-priori seven factor-model was hypothesized based on the attributes considered most essential for the graduating student of the institution. After operationally defining the attributes through extensive literature search, scenarios were constructed to assess them. A 5-point rating scale was used to rate each item on the station. A total 259 students participated in the multiple mini interviews over a period of three days. A training workshop had been arranged for the participating faculty.

**Results:**

The reliability coefficient using Cronbach’s alpha were calculated (range from 0.73 to 0.94), Standard Error of Measurement (ranged from 0.80 to1.64), and item to station-total correlation ranged from 0.43–0.50 to 0.75–0.83. Inter-station correlation was also determined. Confirmatory factor analysis endorsed the results of Exploratory factor analysis in the study revealing a seven model fit with multiple indices of Goodness-of-fit statistics such as Root mean square error of approximation (RMSEA) value 0.05, Standardized root mean square residual (SRMR) value with less than 0.08. All these indices showed that model fit is good. The Confirmatory factor analysis confirmed the multi-dimensional nature of our MMIs and also confirmed that our stations measured the attributes that they were supposed to measure.

**Conclusion:**

This study adds to the validity evidence of Multiple Mini-Interviews, in selection of candidates, with required personality traits for healthcare profession. It provides the evidence for the multi-dimensional structure of Multiple Mini interviews administered with multiple evidences for its internal structure and demonstrates the independence of different constructs being measured.

## 1. Background

Prior academic attainment along with personal qualities or attributes such as empathy, integrity, ethical values, communication, and interpersonal skills are considered essential for student selection in health care professions globally. These attributes are linked to provision of high-quality care and greater satisfaction for both the physician as well as patients. They are also linked to positive medical school performance with improved health care outcomes [1–3]. Considering the high stakes involved, there is a call to employ rigorous mechanisms underlying student assessment and evaluation; such as blueprinting and psychometric analysis, for evaluating the personal attributes as well [4]. There is a caveat though, as these personal attributes are ‘constructs or latent variables which cannot be measured directly and can only be inferred from behavior. Thus, they need to be operationally defined, behaviors reflecting them need to be selected within a vigorous theoretical framework and many validity evidences collected, making the measurement of the attributes difficult and intensive for researchers [5, 6]. Till recently traditional interviews were being used but interviews, even the structured ones are beset with poor reliability and predicative validity. This has led to search for other tools [7, 8].

Multiple Mini interviews (MMIs) provide multiple snapshots of a student’s performance overcoming the issue of context specificity of traditional interviews which has a major influence on its generalizability [7, 9]. MMIs are highly structured and typically consist of a circuit of 4 to 12 stations, of 5 to 8 minutes’ duration and are manned by one or two interviewers [10–12]. The interviewee rotates through these stations, responding to questions or discussing scenarios on issues which they will come across in their life as health care professionals [7, 9]. MMIs have a high acceptability and feasibility leading to an exponential rise in their use. The evidence of its predictive validity, through the course of Undergraduate medical education (UGME) as well as Graduate Medical Education (GME), although form only one institute, is good [10–13]. Literature is however, replete with studies supporting the reliability of MMIs. Some studies have used generalizability studies with adequate G Coefficients when sufficient raters and stations are used [10, 13, 14]. However, there are questions being raised on the use of G coefficient as its statistical assumptions are often not met totally in social science data [15].

Most MMI studies report a single value of Cronbach’s Alpha as the reliability index for the whole MMI, ranging from 0.54 to 0.98 [10–12]. This is understandable as Alpha is the commonest reported measure of reliability in general literature [16]. Serious misconceptions about alpha exist in literature which measures internal consistency (inter-relatedness or similarity of items) of a scale. If the average correlation among items on a scale is high, then the scale is said to be reliable [17, 18]. This high value of alpha is erroneously used as an indication of Uni-dimensionality of scale as well [18]. Uni-dimensionality or homogeneity means that all the items measure the same construct. If this assumption is violated as in the case of multidimensional tests, there can be major underestimation of reliability [18]. Conversely, high single alpha values can still be obtained in multidimensional scales if the test comprises of many items which are similar to some extent [18, 19]. The implication here is that alpha should be calculated only once the dimensionality has been established [19]. For dimensionality the statistical test used is Factor Analysis (FA). Among the FA, Exploratory Factor Analysis (EFA) identifies factors *‘posteriori’* from the data and determines the degree of relationship between items, between items and constructs and also identifies items which do not represent the intended construct and thus should be removed and provides evidence of content validity [6, 17, 20]. In contrast, in Confirmatory Factor Analysis (CFA) the factors and their loading patterns including the number, meaning and associations are decided *‘a-priori*.*’* [6, 15, 18, 20]. Despite the advantages of easiness of availability and use, there is a general lack of researches using FA in instrument development. A systematic review from general education reported that only 16 (16.8%) of the 95 statistical analyses used EFA that met all the pre-requisites [6]. This aberration may be the result of the existing confusion between the ‘traditional’ and contemporary definitions of validity. This contemporary definition conceptualizes validity as a single /unitary concept of ‘Construct Validity’ where multiple evidences from each of the five sources mainly content, response process, internal structure, relationship to other variables and consequences are required to support or refute the inferences drawn from scores, within a specific group and for a particular purpose [5]. The Educational and Psychological Testing made the ‘contemporary’ concept of validity a standard in 1999 [5]. In contrast, the traditional concept of validity comprises of three distinct ‘types’, mainly content, criterion and construct validity [5, 17]. In this traditional concept reliability which indicates, the degree to which the instrument was free from random error at diverse circumstances of the measurement is treated as ‘external’ to validity [17]. This often led researchers to only report reliability measures as sole psychometric evidence. In the contemporary ‘Construct Validity’ concept, reliability is taken as complimentary and the factor structure through FA, reliability measures once the structure is established, Standard Error of Measurement (SEM), item to station-total correlations and inter-station correlations form important internal structure evidences [5, 17, 21].

Unfortunately, the epidemiological and the health sciences researchers including clinicians persist in using the traditional definition of validity [21]. Similarly, the systematic review on MMIs published in 2019 is the only review which analyses the studies in the framework of contemporary framework of validity. Even here only thirty-eight studies out of sixty-four studies reported on internal structure, with twenty studies reporting single measure of alpha for the whole MMI. In this review FA was used by very few studies. This has serious implications. A significant feature of MMIs is the fact that they can be developed in alignment with the institutions’ specific context reflected in their vison and mission statements implication being that psychometric evidence has to be collected in support for MMIs developed in different institutions [12]. Furthermore, MMIs can be developed to intentionally focus on multiple or single attributes. Conversely the personal attributes can be multidimensional constructs for example, self-awareness and communication skills will be required by teamwork/collaboration [22]. However, the scarcity of published studies on the dimensionality of MMIs as well as the contradictory results make it difficult to interpret if MMIs measure a general factor- single construct or one construct per station (i.e. multiple constructs) [13, 14]. Some studies using item response theory have shown unidimensional nature of MMIs [13]. A study in 2019 used EFA and CFA for an eight station MMI where four themes were tested but he results failed to support a multi-dimensional nature of their MMIs [23]. Earlier Cox et al., (2015) [24] had conducted MMI for Pharmacy student and reported multi-dimensional MMIs with seven distinct attributes being assessed on seven stations. The Cronbach alpha ranged from 0.90 to 0.95. They also claimed good evidence of content validity [24]. More concerning is the question mark on whether the MMIs are assessing what they are purported to be assessing [14, 25]. Lemay et al and Hecker et al, using EFA, were able to generate factors but were not able to establish which attributes were exactly being measured by their MMIs [26, 27]. Oliver et al., (2014) using CFA was able to confirm a two factor model but the correlation between the two was very high at .87 making it difficult to interpret if they were assessing different attributes [28].

Very few studies on MMIs are available from Pakistan and fewer still on their psychometric properties. The first Pakistani study found no difference between MMI and structured interviews for assessing non-cognitive attributes except ethics (interview scores mean = 3.04, MMI scores mean = 2.5, P value 0.046) [29]. The second study reports on the faculty perceptions on training before and after MMIs only [30].

A recently published study from Pakistan in 2020 uses generalizability theory with a G coefficient of 0.70. Though it reports multiple measure for reliability it does not use the contemporary definition of validity and also does not report on factor structure [31]. A study published by the authors in 2021 used an *a-priori* multi-dimensional model of MMI and is in fact the only study which reports on multi dimensionality of their MMIs through EFA and alpha for individual stations. Considering the crucial role played by content validity evidences, a strict theoretical framework was followed to provide multiple evidences for content validity and supporting statistical evidence proved that the 9- station multi-dimensional MMIs measured what they were supposed to be measuring [32].

In continuation of our previous work, the present study is designed to provide psychometric evidence of internal structure for MMIs. Our specific objective is to provide support of the multi-dimensional nature of our, a*-priori* hypothesized MMIs, through CFA. We aim to provide multiple evidences for the internal structure including SEM and item-to-station total correlation. Our study highlights the link between content and internal structure evidences of the constructs, thus establishing that our MMIs measure what they were intended to be measuring.

## 2. Methods

The present psychometric study reports on MMIs administered in 2017 at a private medical college in Islamabad, Pakistan, after taking ethical approval from the Institutional Review Board (IRB). Thirty-five faculty members were trained, who assessed 259 students in a seven station MMI, over three days. The study duration was September 2017- February 2018 and participants were recruited during this period. Study comprised the cohort of students that took admission in 2017. To ensure familiarization of students with test format the applicants were informed via telephonic message about MMI’s being conducted in place of traditional interviews. The process of MMI’s was explained and students’ queries were answered by two members of the respective department in this telephone conversation. The students were briefed again by the department staff on the day of MMI and written informed consent was taken from them. Data was entered using SPSS version 20.

### 2.1 Introducing MMIs for UGME—2013

**Fig 1:**
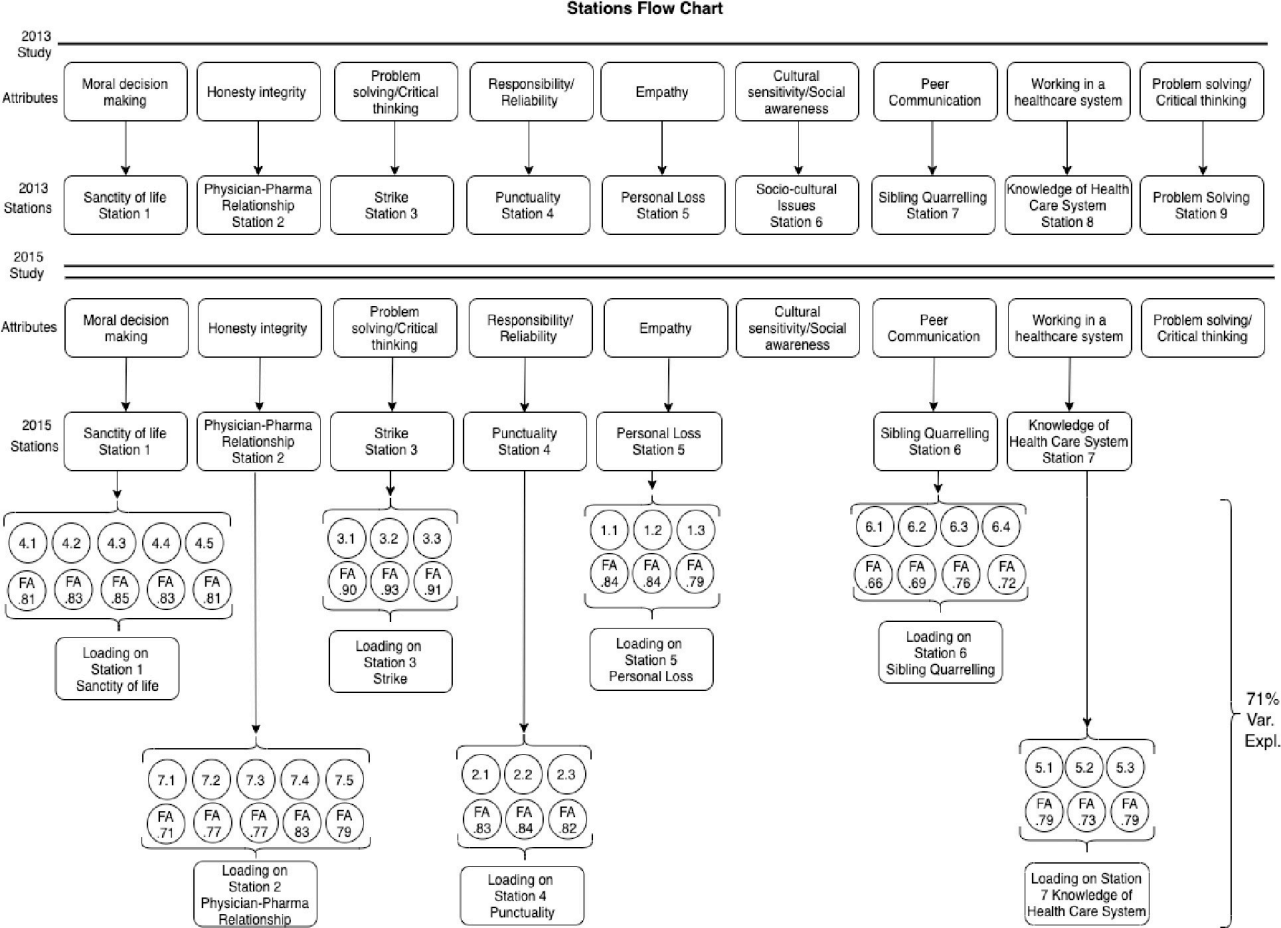
Flow chart showing nine station MMI of 2013 and seven station MMI of 2017 with number of items for each station and factor loadings.

A policy level decision had been made in our institution, to introduce MMIs for admissions in Undergraduate Medical Education (UGME) Program in 2013. An *a-priori* hypothesized nine-factor-model was realized for the MMIs where each of the nine stations was intended to assess a distinct attribute. A core team comprising of educationists and senior faculty members, developed a blueprint of nine attributes aligned to the institutional vision after extensive literature search. To ensure content validity, each attribute was operationally defined and behaviours reflecting these constructs were carefully selected from literature. A 5-8-line scenario served as a trigger for the station and had 3–5 items testing the underlying attribute. Each of these items was rated on a 5-point rating scale. A whole day training workshop was conducted with the dual purpose of training the faculty on conducting MMIs as well as for pilot testing the stations. First year students from our dentistry program were involved in this exercise. The cycles were repeated and inter-rater reliability was calculated till a coefficient of .8 was reached. At the end of training, feedback was taken from faculty and students and their input was used to modify the scenarios. Finally, 365 students rotated through nine stations of MMI, over a course of six days and were assessed by 9 different assessors. Cronbach’s alpha ranged from 0.64 to 0.98, SEM ranged from 3.41% to 8.97% and item-total correlation ranged from 0.53 to 0.96. The results of EFA showed a ten-factor structure instead of nine with all the stations loaded onto their factors except the station on empathy which loaded onto two factors as given in Fig 1.

### 2.2 Modifications in MMIs- 2017

Modifications were made in the MMIs for 2017 cohort, based on feedback from faculty, students, and other stakeholders. This year, stations were reduced to seven from the original nine. In 2013 the attribute ‘critical thinking’ was evaluated on two stations. It was decided to reduce it to one station to reduce administrative burden and the station on ‘cultural sensitivity’ was omitted as the majority of students had performed poorly in it. The faculty felt that the high school students did not have enough exposure or maturity to discuss such social issues. A foundational module on ethics named ‘Foundation of medical practices’ was rolled out for upcoming classes on faculty’s suggestions. The station on empathy was rewritten as it had loaded onto two factors in our earlier study. A training workshop was conducted for the participating faculty on the lines of 2013 cohort and the rewritten scenario was included. Other stations used previously constructed scenarios as triggers.

### 2.3 Factor analysis for determining the factor structure

In our study an *a-priori* seven-factor model was hypothesized after extensive literature research for establishing the theoretical meaning of the constructs and blueprinting. This was followed by EFA the Kaiser-Meyer-Olkin (KMO) for sample adequacy for each variable in the model and for the overall model. The KMO value was found 0.85, indicating that sample was adequate, and Bartlett’s Test of Sphericity gave a p-value of <0.001. Item-wise data for all stations was used for EFA. All the prerequisites for conducting EFA like large sample was fulfilled. Correlation coefficients over 0.30 were identified as the benchmark. Additionally, it is expected that at least two or three variables must load on a factor so it can be given a meaningful interpretation. To fulfil this requirement all the attributes were tested by a minimum of three items. A total of 26 items were used to assess the seven stations. Since more than one approach is recommended for factor extraction, the Scree test and the cumulative percent of variance extracted were also assessed. Fig 1 shows the attributes aligned to nine stations of MMI of 2013 and to seven stations of MMI 2017 with number of items for each station and factor loadings.

Cronbach’s alpha coefficient for each station was determined after EFA confirmed the factor structure. In order to provide multiple evidences for our internal structure, Item- station-total correlation which assesses the degree to which each item on a MMI station score correlates to the total station score and inter-station correlation was determined. For determining the precision of the observed score, SEM was calculated. Furthermore, CFA was conducted for the confirmation of our hypothesis and different measures such as chi-square test, RMSEA, SRMR, comparative fit index (CFI), tucker-lewis index (TLI) for the goodness of fit were used to assess how well the data fit for the evaluation. The latest versions of SPSS (Version 27) and R–Software was used for the above parameters corresponding to the reliability and validation procedure.

## 3. Results

### 3.1 Descriptive statistics

Total 259 applicants (male 127 and female 132) participated in the MMI on the test day. Out of these, 170 belonged to urban areas and 89 were from rural area. Convenience sampling method was used to select the sample for the study and all the participants were included in the study. MMI stations with the number of items and mean score (%) with Standard Deviation (SD), SEM and Item-Total Correlations are shown in Table 1.

**Table 1:** Constructs with their reliabilities.

Constructs	No. of Items	Mean ±SD	95% CI	Cronbach alpha	SEM	Item-Total Correlation (Range)
**Health-Advocacy-Knowledge of healthcare system**	3	3.49± 0.05	3.40–3.59	0.83	0.97	0.57–0.62
**Empathy- Personal Loss**	4	3.55± 0.05	3.46–3.64	0.83	0.95	0.59–0.66
**Critical thinking/Problem solving -Strike**	5	2.91± 0.07	2.78–3.04	0.92	1.31	0.75–0.83
**Responsibility/ reliability- Punctuality**	3	2.97± 0.05	2.86–3.07	0.90	1.15	0.54–0.81
**Ethical Dilemma-Sanctity of life**	3	3.47± 0.05	3.37–3.57	0.73	0.80	0.43–0.50
**Honesty integrity-Physician-pharma relationship**	3	3.06± 0.04	2.98–3.13	0.74	1.29	0.30–0.52
**Communication Skills**	5	3.60± 0.04	3.52–3.68	0.86	1.64	0.47–0.66

The mean scores ranged from 2.91± 0.07 for the construct related to Responsibility/ reliability Punctuality to 3.60 ± 0.04 for the station measuring communications skills. The item-total correlations ranged from 0.30–0.52 to 0.75–0.83.

### 3.2 Internal structure evidence

**Fig 2:**
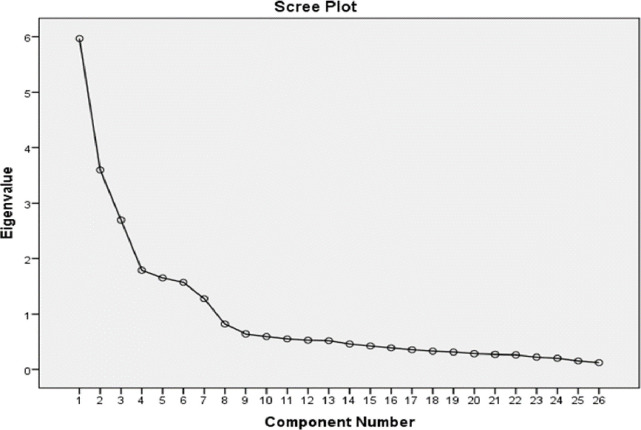
Scree plot for 2017 cohort.

Twenty-six items loaded onto seven-factor model after six iterations (Fig 2 & Table 2). The rotated factor analysis revealed that all seven stations were completely aligned with their items and were measuring underlying constructs. The seven factors cumulatively accounted for 71% of variance with variance of station assessing knowledge of health care system being highest (22.94%) and the variance of communications skills lowest at 4.91% (Table 2).

**Table 2:** Factor rotation matrix table of exploratory factor analysis (2017 cohort).

Factor/	1	2	3	4	5	6	7
Station Name	Station 4	Station 7	Station 3	Station 2	Station 1	Station 6	Station 5
	Communication skills	Critical thinking/ problem solving	Honesty integrity-	Responsibility/ reliability	Health-Advocacy	Empathy	Sanctity of life
**Factor Loadings of Station Items**	.810	.712	.901	.835	.842	.694	.793
	.835	.771	.930	.847	.840	.760	.739
	.858	.779	.911	.824	.790	.723	.791
	.832	-	-	-	-	-	-
	.817	-	-	-	-	-	-
**% Variance accounted for**	22.94	13.83	10.36	6.88	6.35	6.04	4.91

In this EFA all the stations were completely aligned with their items and were measuring underlying constructs including the station on empathy which had split onto two stations in 2013. Scree plot for this factor loading is also given in Fig 2. Reliability was measured as Cronbach’s alpha for individual stations and ranged from 0.73 to 0.92. Cronbach’s alpha, SEM and Item- station-total correlation are given in Table 1. The total alpha was also calculated which came out to be 0.85and it can be seen that the majority of individual stations have alpha values which are much higher than single alpha of cohort. (Table 1). Table 3 provides the inter-station correlation of the seven stations further confirming that each station was assessing a discrete attribute.

**Table 3:** Inter-station correlation.

Station Details	Station Total 1	Station Total 2	Station Total 3	Station Total 4	Station Total 5	Station Total 6	Station Total 7
**Station Total 1**	1	0.296*	0.222*	0.178*	0.216*	0.216**	0.365**
**Station Total 2**		1	0.267*	0.145**	0.145**	0.105*	0.262*
**Station Total 3**			1	0.006*	0.13**	0.026*	0.117*
**Station Total 4**				1	0.227**	0.409**	0.101*
**Station Total 5**					1	0.345*	0.253*
**Station Total 6**						1	0.31*
**Station Total 7**							1

**Correlation is significant at the 0.01 level (2-tailed)

*Correlation is significant at the 0.05 level (2-tailed)

### 3.3 Confirmatory factor analysis

CFA was conducted to examine the relationship between factors and their loadings. Given our objectives and the results from EFA, we first assessed the goodness of fit of our CFA model according to Hu and Bentler cut-off criteria [33]. According to these cut-points, these measures include comparative fit index (CFI >.95), Tucker-Lewis index (TLI > .95), Standardized root mean square residual (SRMR < .09) and root-mean-square errors of approximation (RMSEA ≤ .05). In this model we observed CFI = 0.96, TLI = 0.95, SRMR = 0.08 and RMSEA = 0.05. All these measures showed good fit and indicate that model is adequate. Observations with missing values were excluded. Standardized coefficients were used and the estimation model was maximum likelihood. The CFA function fixes a factor loading for each factor to be 1 and estimates the rest of factor loadings. Different from EFA, CFA also tested the significance of the factor loadings based on a z-test. Table 4 shows the coefficients of the hypothesized relationships, together with their z-values, standard errors and p-value for the tested model. Instead of fitting a factor loading to be 1, we can also fix the factor variances to be 1. Coefficients in Table 4 are observed significant at 0.05% level of significance. All the covariance’s are also observed significant.

**Table 4:** Standardized coefficients and associated data.

Items	Coefficients	Standard error	Z-value	p-value
*Health-Advocacy-Knowledge of healthcare system*				
Item 1	0.301	0.038	7.887	0.000
Item 2	0.229	0.039	5.892	0.000
Item 3	0.375	0.044	8.520	0.000
*Empathy- Personal Loss*				
Item 1	0.259	0.035	7.376	0.000
Item 2	0.226	0.038	5.894	0.000
Item 3	0.386	0.045	8.679	0.000
*Critical thinking/Problem solving—Strike*				
Item 1	0.339	0.038	8.888	0.000
Item 2	0.107	0.030	3.584	0.000
Item 3	0.310	0.038	8.185	0.000
*Responsibility/ reliability—Punctuality*				
Item 1	0.350	0.038	9.332	0.000
Item 2	0.341	0.038	8.905	0.000
Item 3	0.299	0.034	8.906	0.000
Item 4	0.278	0.031	8.893	0.000
Item 5	0.329	0.035	9.344	0.000
*Ethical Dilemma-Sanctity of life*				
Item 1	0.636	0.072	8.792	0.000
Item 2	0.497	0.069	7.211	0.000
Item 3	0.566	0.081	6.961	0.000
*Honesty integrity-Physician-pharma relationship*				
Item 1	0.395	0.043	9.130	0.000
Item 2	0.268	0.032	8.470	0.001
Item 3	0.425	0.046	9.296	0.002
Item 4	0.445	0.051	8.755	0.000
*Communications Skills*				
Item 1	0.309	0.033	9.464	0.000
Item 2	0.316	0.032	10.023	0.000
Item 3	0.326	0.033	9.789	0.000
Item 4	0.214	0.026	8.100	0.000
Item 5	0.229	0.028	8.193	0.000

All Coefficients are significant at 0.05 level

## 4. Discussion

Our present study is designed to provide psychometric evidence for the multi-dimensional nature of MMIs conducted to assess the personal attributes of students entering into UGME program, in resource restricted settings like Pakistan. Our study provides multiple evidences for the internal structure and most importantly it provides evidence that our MMIs measure what they were intended to measure. To the best of our knowledge this is the only such study which utilizes the contemporary definition of validity and caters to the above mentioned features.

MMI were developed by Eva et al., (2004) [9] to assess applicants on multiple attributes, thought to be relevant to the future competent health care practice so as to overcome the issue of context specificity [7, 9]. Our analysis showed that student performance was acceptable on stations testing critical thinking, problem-solving, and communication skills, working in healthcare systems, honesty, responsibility, and reliability as shown by the mean score of the stations.

Our MMIs were developed utilizing evidence based practices underlying assessment of students like blueprinting and psychometric evidences including validity and reliability to support student selection methods, something which was missing for interviews, structured or traditional [4, 7]. MMIs are well supported by predictive validity studies [10, 11]. However, majority studies are only reporting single value of alpha. They also fail to provide evidence for the link between the structure of the construct informed by the literature and the empirically derived factor structure. This is found to be missing in general literature reporting measurement of psychological construct [6]. To address this gap, diligent efforts were made to define the constructs and behaviors reflecting them were collected carefully, within a vigorous theoretical framework, by our team. This was followed by expert review and pilot testing of the items thus ensuring the content validity.

Our study then successfully employed both EFA and CFA to provide empirical evidence that the set of identified items measured the same construct and to confirm that the items function as intended. In our initial study majority items had loaded onto their factors [32], but one station had split into two factors leading to a 10 factor structure for the *a–priori* hypothesized 9 station MMI leading to the conclusion that station was not measuring the desired construct. This led to revision and modification of the station for 2017 cohort. In our present study EFA has resulted in all stations loading onto their respective factors providing good evidence of content validity and stability of our results. Our stations formed single factors with loadings ranging from 0.694 to 0.911 (Factor loadings < 0.3 are suppressed). Cronbach’s Alpha was calculated for individual stations and ranged from 0.73 to 0.94 reflecting the inter-relatedness of the items assessing each construct. Our reliability estimates are similar to the published estimates [10–12]. It is important to note that calculating alpha before establishing the dimensionality can lead to erroneous high or low values of alpha [15, 17]. To support our argument, single alpha for the whole MMI was also determined and was found to be 0.85. As seen in results many of our stations had alpha values much higher than the single alpha of the cohort further reflecting that alpha will give erroneously low values if heterogeneous scales are considered as homogenous and single alpha is computed. We used CFA in our study to confirm the multi-dimensional nature of our MMIs. All the estimates were found to be significant. CFA endorsed the results of our EFA revealing a seven model fit with multiple indices of Goodness-of-fit statistics such as Root mean square error of approximation (RMSEA) value 0.05, Standardized root mean square residual (SRMR) value with less than 0.08 showing the good fit of our model.

Although FA provides empirical evidence that all the items represent the same construct and that there are sufficient and appropriate items representing all the important aspects of the construct with no irrelevant items, it is often criticized as being inefficient [34]. Our results lead us to believe that FA are easily available and administered tests which when combined with carefully collected multiple evidences for content and internal structure, can provide assurance that, the researcher is correctly interpreting the results as representing the construct the instrument purports to measure [6]. In our study, additional evidence for internal structure were garnered from item-total correlation which ranged from 0.53 to 0.96. The high internal consistency and item-total correlations for each station validated that the content of each station was assessing the desired *a-priori* hypothesized constructs. The inter-station correlation was minimal which showed that all our stations were measuring separate constructs further providing evidence to the multi-dimensional nature and appropriateness of measurement.

Our study shows the importance of moving to contemporary definition of ‘construct validity’ with easy and simple tests like FA for ensuring psychometric rigor which can help resource restricted institutions of countries like Pakistan to implement this important student selection method for student selection with the confidence that necessary constructs representing the required attributes are being assessed.

## 5. Conclusion

Our study shows that our MMIs measured the intended attributes and were multi-dimensional in nature. It provides evidence that first establishing theoretical basis of the constructs through literature search followed by EFA confirms the relationships between the items and constructs. Finally, CFA provides the evidence that our MMIs measured what they were supposed to measure. Our study also provides multiple evidences for internal structure and establishes the need for determining the factor structure before calculating the reliability so as to avoid erroneous interpretations of alpha. It provides an impetus to researchers that studies on MMI must be developed keeping in mind the need to move from traditional concept of validity to contemporary concept to the extent possible and to develop a body of literature where they can have confidence in the conclusions drawn from these measures.

## 6. Limitations of the study and recommendations

This study reported results of MMI only from a single institution. Since these MMIs were developed relevant to the institutional context and requirements, the results may not be generalizable. CFA was carried out on the same sample as EFA. Though it is not a recommended practice but we wanted to provide multiple sources of evidence that our MMIs were multidimensional. We did not have to force the models to fit or made any post hoc changes otherwise we would have divided the sample in half and conducted EFA on one half and CFA on the other to avoid an artificial good model fit. Such a situation however did not arise.

We were also unable to provide information of interviewee demography in detail except its gender or any possible links to their performance. Despite such rapid rise in the use of MMIs globally and the huge literature in its support, we would like to mention that no tool is without its limitations. If majority of applicants selected by MMI are those who possess certain specific personal traits, then the diversity amongst applicants might be reduced, which in turn might limit professional and personal growth of future physicians.
